# Innate immune responses in hepatitis B virus (HBV) infection

**DOI:** 10.1186/1743-422X-11-22

**Published:** 2014-02-07

**Authors:** Aurelia Busca, Ashok Kumar

**Affiliations:** 1 Departments of Pathology and Laboratory Medicine, University of Ottawa, 451 Smyth Road, Ottawa, Ontario K1H 8M5, Canada; 2 Biochemistry, Microbiology and Immunology, and Infectious Disease & Vaccine Research Centre, Research Institute, Children’s Hospital of Eastern Ontario, University of Ottawa, Ottawa Ontario, Canada

**Keywords:** HBV, Innate immunity, Cytokines, Interferon response, TLRs, RIG-I

## Abstract

Hepatitis B virus (HBV) infection has a low rate of chronicity compared to HCV infection, but chronic liver inflammation can evolve to life threatening complications. Experimental data from HBV infected chimpanzees and HBV transgenic mice have indicated that cytotoxic T cells are the main cell type responsible for inhibition of viral replication, but also for hepatocyte lysis during chronic HBV infection. Their lower activation and impaired function in later stages of infection was suggested as a possible mechanism that allowed for low levels of viral replication. The lack of an interferon response in these models also indicated the importance of adaptive immunity in clearing the infection. Increased knowledge of the signalling pathways and pathogen associated molecular patterns that govern activation of innate immunity in the early stages of viral infections in general has led to a re-evaluation of the innate immune system in HBV infection. Numerous studies have shown that HBV employs active strategies to evade innate immune responses and induce immunosuppression. Some of the immune components targeted by HBV include dendritic cells, natural killer cells, T regulatory cells and signalling pathways of the interferon response. This review will present the current understanding of innate immunity in HBV infection and of the challenges associated with clearing of the HBV infection.

## Review

### Introduction

Hepatitis B virus (HBV) is a noncytopathic, hepatotropic virus of the *Hepadnaviridae* family that causes variable degrees of liver disease in humans. Infection with HBV can be either acute or chronic; while adult infections have a relatively low rate of chronicity (around 5%), neonatal infections usually have a high persistence rate [1]. Chronic infection is mostly asymptomatic, but HBV carriers are at risk of developing life threatening cirrhosis and later on hepatic carcinoma [2]. Despite the availability of a prophylactic vaccine, HBV is estimated to infect around 400 million people worldwide, with endemic areas is Asia and Africa.

HBV replication itself is not directly cytotoxic to cells, as seen in the large numbers of asymptomatic HBV carriers who have minimal liver injury, despite ongoing intrahepatic replication of the virus. The long-term aim in the treatment of these patients is to prevent the development of cirrhosis and hepatocellular carcinoma [3]. The immune responses to HBV antigens are responsible both for viral clearance during acute infection and for disease pathogenesis. In infected humans, viral clearance follows the development of a vigorous immune response associated with acute, self-limited inflammatory liver disease (acute viral hepatitis). Immune responses involved in viral clearance comprise both humoral and cellular immunity. Major-histocompatibility-complex (MHC) class II–restricted, CD4+ helper T cells contribute to generation of antibodies against viral envelope antigens that clear circulating virus particles. MHC class I–restricted, CD8+ cytotoxic T lymphocytes eliminate infected cells [4].

Experimental approaches to HBV pathogenesis are hampered because the host range of HBV is limited to man and chimpanzees. The functional receptor for HBV has recently been identified as sodium taurocholate cotransporting polypeptide (NTCP), a molecule implicated in bile salts transport [5]. A lot of experimental data has come from studies with hepatitis B virus transgenic mice that harbour viral antigens and replication-competent viral genomes in the liver. These mice harbour HBV genes in their germ-line DNA, therefore they are tolerant to HBV infection and do not develop significant liver injury despite high levels of replication [6]. These studies have shown the importance of cytotoxic CD8+ lymphocytes (CTLs) in controlling viral replication, as adoptively transferred virus-specific CTLs can abolish HBV gene expression and replication in the liver of HBV transgenic mice by causing acute liver injury [7]. Moreover, transgenic mouse studies have shown that liver disease has an immunological basis rather than being virus mediated, with CTLs also playing a central part in this process [8].

One way to overcome the inherent tolerance of HBV-transgenic mice to viral antigens was to use human hepatocyte chimeric mice, where human hepatocytes repopulated the liver of uPA/SCID mice, making it susceptible to HBV infection [9]. This model is a valuable tool for studying viral mutants and various therapeutic agents; however, the lack of human immune cells also limits the study of inflammation and immunity.

Earlier studies in animal models failed to detect innate immune responses in acute stages of HBV infection, which led to a general consensus that HBV doesn’t induce innate immunity during natural infection. Despite of the development of a successful prophylactic vaccine, a lot of immunpathological processes remain unknown, especially with respect to chronic HBV infection. In this setting, virus-specific CD8+ T are present in the liver, but are impaired functionally or quantitatively so that they are unable to clear the infection and likely contribute to disease pathogenesis [10]. This has rekindled the interest in the innate immune responses and their impact on viral biology.

It is now thought that host immune responses to viral antigens and to viral replication in infected hepatocytes are the main determinants of hepatocellular injury and HBV pathogenesis. This review will focus on the determinants of innate immunity in controlling viral replication and their contribution to viral pathogenesis, with respect to both acute and chronic infection.

In general, innate immunity is important in controlling infection immediately after contact with the pathogen, in order to limit the spread of the infection and to initiate efficient development of an adaptive immune response. The innate immune system is activated by pathogens by using pattern-recognition receptors (PRRs) that recognize specific structures on pathogens, such as double-stranded RNA and bacterial wall components. The most important PRRs in viral infections are Toll-like receptors (TLRs), RNA helicases, such as retinoic acid inducible gene I (RIG-I) and melanoma differentiation associated gene 5 (MDA5), and double stranded RNA-dependent protein kinase (PKR) [11].

Of the TLRs, TLR9 detects viral DNA, TLR7 and TLR8 identify viral single-stranded RNA, while TLR3 recognizes viral double-stranded RNA [12]. Following recognition of virus associated molecular patterns by cellular PRRs, recruitment of their distinct adaptor proteins will sequentially activate signalling cascades to induce cytokine production in virus-infected cells.

The early phase of viral infections is mainly characterized by the production of cytokines, type 1 interferon (IFN)-α/β, and the activation of natural killer (NK) cells. Production of type 1 IFNs can be triggered directly by virus replication through cellular mechanisms that detect the presence of viral RNA or DNA. The main sources of IFN-α/β are infected cells and plasmacytoid dendritic cells, whereas IFN-γ is produced primarily by NK and NKT cells [13].

### Interferon response

Earlier studies have evaluated whether HBV has the ability to stimulate the interferon pathway of the innate immune response during the early stage of infection. Data from patients and animal models have shown that there is a delay between HBV inoculation and efficient replication. Both HBV-DNA and HBV antigens become detectable around 5 weeks post-infection, after which viral titers have a logarithmic expansion phase and HBV infects most hepatocytes [14,15]. While it is tempting to speculate that this initial lag of replication is a result of efficient control of the virus by the innate immune response, animal studies have indicated that this is not the case, but rather that somehow the virus manages to evade being recognized. In contrast to the immune response in HCV infection, where expression of a large number of IFN-regulated genes was rapidly induced in the liver of HCV-infected chimpanzees [16], the same group has shown that HBV did not cause any change in genes associated with innate immune responses during acute HBV infection of chimpanzees [17]. The authors concluded that HBV did not induce an intrahepatic innate immune response that could be detected in the infected animals because it acts like a stealth virus early in infection, remaining undetected and spreading until the onset of the adaptive immune response several weeks later. However, the mechanisms by which HBV accomplishes this task are less understood.

Interestingly, HBV replication is sensitive to the suppressive effects of interferons in studies using the transgenic mouse model or hepatoma cell lines. HBV was shown to replicate in IFNγ KO and IFNα/β receptor KO mice at levels higher than those observed in control mice, implying that baseline levels of these cytokines control HBV replication in the absence of inflammation [18]. Also, expression of adaptor proteins TRIF and MyD88 (TLR adaptor proteins) and of IPS-1 (RIG-I/MDA5 adaptor protein) in human hepatoma cells was enough to limit HBV replication by reducing the steady-state levels of viral mRNAs, in a manner dependent on NF-κB transcription factor [19]. The importance of TLR receptor signalling in controlling HBV replication was confirmed in another study showing that a single intravenous injection of ligands specific for TLR3, TLR4, TLR5, TLR7, and TLR9 was efficient to inhibit HBV replication in a noncytolytical and IFNα/β dependent manner in HBV transgenic mice [20].

Another characteristic of the interferon response in HBV infection is that CD8+ T cells seem to control the viral replication through an IFN-γ dependent mechanism rather than direct cell killing of infected hepatocytes [15]. The importance of noncytolytic T cell functions was shown in a study where the onset of viral clearance in HBV infected chimpanzees was tightly associated with the appearance of virus-specific T cells and mRNA in the liver, before the onset of hepatocyte destruction and clinical hepatitis [21]. These results suggest that direct cytological cell killing and IFN-γ secretion are functionally distinct mechanisms of CD8+ T cells that account for control of hepatocyte destruction and viral replication, respectively.

### Cytokines

Other cytokines have also been reported to control HBV replication in the transgenic mouse model, including interleukin 12 (IL-12) [22] and IL-18 [23], the effect being mediated by induction of IFN-γ and IFNα/β respectively. Injection of HBV transgenic mice with CD40 ligand to induce maturation of antigen presenting cells lead to recruitment of macrophages, dendritic cells and NK cells to the liver and induced secretion of TNF-α, IFN-γ, and IFNα/β, which ultimately resulted in inhibition of HBV replication [24]. In a primary human hepatocyte model, Hosel et al. showed that briefly after infection, liver macrophages recognize HBV and induce production of TNF-α, IL-6, IL-8 and IL-1β. However, the ability to control viral replication was attributed to IL-6 via NF-κB activation [25].

Another major antiviral cytokine is TNF-α, which was initially shown in HBV chimpanzee model to contribute to the antiviral effects of CTLs alongside IFN-γ, although initially the source of cytokines was unknown [7]. In the HBV transgenic mouse model, TNF-α was secreted by antigen presenting cells following activation as part of the inflammatory cascade during acute infection [24]. Non-parenchymal liver cells like Kupfer macrophages and liver sinusoidal endothelial cells are also part of the innate immune response due to their ability to respond to TLR signalling by producing various cytokines, including TNF-α and IFN-β [26]. A recent report has also shown that TNF-α and IL-1β can restrict HBV replication via activation of the NF-κB signaling pathway [27]. Early elevation of IL-10 and IP-10 has also been linked with better outcome during HBV vaccination [28].

Taken together, these results suggest the existence of a functional innate antiviral mechanism in hepatocytes that is associated with favourable outcome in both acute and chronic infections. Its activation under certain conditions may also be used for therapeutic purposes. Activation of innate immunity to induce interferon production but not killing of infected hepatocytes may also represent a new strategy for the treatment of chronic HBV infection, in the absence of cytological cell death associated with the activation of CD8+ T cells.

### Dendritic cells

DCs are major players in immune responses, due to their ability to process foreign antigens and present them to effector cells. The presence of HBV or HBsAg during cytokine induced maturation of dendritic cells resulted in a more tolerogenic phenotype, as shown by a decreased T-cell stimulatory capacity [29]. Other recently reported targets for HBV are plasmacytoid DCs (pDCs), which are major components of the antiviral immune response, due to their ability to secrete type 1 IFNs. However, HBV did not activate human pDC in vitro, but HBV virions and HBsAg were able to abrogate TLR7- and 9-induced IFNα gene transcription by direct binding TLR9-triggered pDC [30]. Also, TLR9-activated pDCs from HBV infected patients were impaired in their ability to induce cytolytic activity of NK cells [31]. These studies indicate that the virus exerts an immune regulatory effect that may directly contribute to its persistence during chronic infection.

### T regulatory cells

The immuno-modulatory properties of HBV are also reinforced by studies that show changes in the T regulatory cells (Tregs) population in HBV chronically infected patients. CD4 + CD25+ Tregs normally maintain immune tolerance against self and foreign antigens, due to their ability to produce IL-10 and TGF-β. During chronic HBV infection they seem to have a deleterious effect, because they increase in number compared to acute infection or healthy controls and this increase correlates with the viral load, suggesting a direct effect on viral replication [32,33]. Generation of Tregs is also a mechanism by which pDCs contribute to immunological tolerance in healthy individuals [34]. This effect also contributes to the immune suppression that characterizes chronic infection, since pDCs from patients with chronic HBV infection were able to generate a higher proportion of Tregs compared to healthy controls [35].

Moreover, Tregs were capable of suppressing the proliferation and IFN-γ production of autologous peripheral blood mononuclear cells mediated by HBV antigen in vitro [36], and increased numbers of Tregs were found in the peripheral blood of patients with HBV related liver failure [37], suggesting that they play an active role in the strategy employed by HBV to downregulate effector immune responses.

Other evidence to support active immunosuppressive strategies employed by HBV includes its ability to affect the production of IL-10, a cytokine with known immunosuppressive effects. HBcAg was able to induce significantly higher IL-10 levels in peripheral blood mononuclear cells from patients with chronic HBV infection compared to those from healthy controls [38].

### Monocytes, natural killer cells and NK T cells

In this setting of decreased activation of cytotoxic T cells, there is a continuous stimulation of other cell types that may explain the persistence of chronic infection and subsequent liver inflammation that characterize chronic HBV infection. The mechanism of chronic inflammation involves recruitment of various cell types of the innate immune system, including monocytes/macrophages and NK cells [39]. Non-classical proinflammatory monocytes of the CD14^low^CD16^+^ phenotype have increased secretory functions and the ability promote fibrosis in other inflammatory diseases and infections [40]. Their expression is increased in both the peripheral blood and liver of patients with chronic HBV infection, where they positively correlate with liver inflammatory damage [41,42], suggesting their active role in this process.

The lack of type 1 IFN responses in the setting of in vivo infection seems surprising, given the fact that the majority of infected individuals are able to resolve the infection. This indicates that the viral replication may be controlled by other arms of the innate immune system.

Natural killer cells (NK cells) are the main effector population involved in innate immune responses to viral infections. They exert antiviral activity through direct cytotoxic effect or through the production of immunoregulatory cytokines (IFN-γ, TNF-α, TGF-β, IL-10, etc). They are identified by flow cytometry by the expression of CD56 and lack of T cells marker CD3. CD56+ NK cells also express the FcγIII receptor CD16, which allows them to mediate antibody-dependent cell-mediated cytotoxicity [43]. NKT cells comprise a T cell subpopulation characterized by expression of surface markers of NK cells together with a T cell receptor that recognizes glycolipids associated with MHC class I-like CD1 molecules [44].

Accumulating evidence indicates that NK cells are major determinants of the clinical outcome following HBV infection. A study performed on two seronegative blood donors who became positive for HBsAg and HBV DNA without evidence of liver pathology, indicating successful containment of infection, were monitored throughout early stages of infection. Their results indicate an early activation of human innate immune system consisting of CD56^+^CD3^–^ NK and CD56^+^CD3^+^ NKT cells. These two cell types were promptly activated before maximal HBV DNA elevation and HBV-specific T cell responses [45]. This study suggested that early and efficient induction of innate responses control the infection by non-cytolytic mechanisms rather than by hepatocyte lysis and may also contribute to timely induction of adaptive responses. Moreover, augmentation of IFN-γ and IL-15 producing CD56^+^ NK cells is also an immunomodulatory effect of pegylated - IFN-α treatment during chronic HBV infection [46].

These results are in contrast to those observed the chimpanzee model [17] and may reflect differences between the two hosts. Studies on NK cell function early in natural human infection are rare because the incubation period of HBV infection is always asymptomatic and therefore difficult to study.

In a study looking at the response of NK and NKT cells following vaccination with HBsAg, there was an increased activation and IFN-γ production in NK and NKT cells following ex vivo incubation with recombinant HBsAg in responders to vaccination. These effects were not observed in non-responders to the vaccine, suggesting that the two cell types play an important role in the immune response following hepatitis B vaccination by also contributing to a robust adaptive immune response [47]. The ability of NKT cells to control viral replication was also confirmed in vivo. Upon activation of NKT cells with a glycolipid, HBV replication was abolished and IFN-α, β and - γ were detected in the liver of HBV transgenic mice [48]. Moreover, these effects persisted in the absence of conventional CD4+ and CD8+ T cells [48], suggesting that the innate immune response has the potential to contain the virus during natural HBV infection.

However, activation of NK and NKT cells has also been linked to liver injury in HBV transgenic mice, due to their ability to secrete proinflammatory cytokines in the hepatic microenvironment [49]. The involvement of NK activation in liver injury was not observed in human studies. On the contrary, one study comparing NK cell function in patients with chronic HBV infection with those from chronic HCV infection and healthy controls showed a more pronounced functional defect in chronic HBV infection than in the other two groups [50]. One recent study showed that NK numbers and cytotoxic function are maintained in peripheral blood of patients with chronic HBV infection compared to healthy controls, but their activation and cytokine production abilities (IFN-γ and TNF-α) are impaired [51]. Overall, these studies indicate that the main function of NK and NKT cells is to limit viral replication via cytokine production rather than direct cytotoxicity and that function is compromised during chronic HBV infection, which may contribute to viral persistence.

Although the exact contribution of NK and NKT cells to chronic inflammation that characterizes chronic hepatitis B is yet to be established, the current literature suggest that strong innate responses are associated with subsequent adaptive immune activation and control of viral replication in the absence of hepatocyte lysis. Inefficient activation of the innate pathways gives rise to a lower, but persistent proinflammatory activity that cannot clear the infection and has deleterious effects on the liver microenvirnment.

### Viral strategies to counteract innate immune responses

Recent developments in our understanding of the HBV infection have shown that the virus is not just a stealth pathogen, but that it employs active mechanisms to avoid recognition by the innate immune system. This may explain previous observations on its apparent inability to be detected by PRRs.

As mentioned, RNA helicases, such as RIG-I and MDA5 are important PRRs responsible for recognition of viral RNAs produced during viral infection and they represent targets for immunosupression during HBV infection [52]. Viral evasion strategies involve targeting IFN-regulatory factors (IRF) which culminate in IFN production. HBV polymerase was reported to impair antiviral innate immune responses by inhibiting IRF activation in response to TLR3- and RIG-I- induced pattern recognition receptor signalling in human hepatoma cell lines [53-55].

Other HBV proteins were shown to target adaptor proteins of the RIG-I pathway.

Regulatory HBx protein binds to beta interferon promoter stimulator 1 (IPS-1) and targets it for inactivation. The consequence of HBx binding to IPS-1 is inhibition of double-stranded DNA mediated IFN-β activation in hepatoma cell lines [56]. Human cell lines studies have also indicated that adaptor protein mitochondrial antiviral signalling (MAVS) is another target for HBx. By promoting degradation of MAVS, induction of IFN-β through the RIG-I pathway is prevented [57].

Another pathway that HBV uses to attenuate the antiviral responses of innate immunity is the TLR system. TLR receptors recognize nucleic acid sequences in degraded viral particles in endosomal or lysosomal cell compartments. TLR triggering initiates signal transduction pathways that promote dendritic cell maturation, production of key cytokines such as type I IFNs and initiation of adaptive immunity [58].

TLR3- and TLR4-activated murine nonparenchymal liver cells are part of the innate immune response following infection and also possess the ability to suppress HBV replication via IFN-β induction [59]. The same group has also shown that the virus has evolved mechanisms to counteract this effect. HBV virions or viral antigens such as HBsAg and HBeAg abrogated TLR-induced antiviral activity, by suppressing IFN-β production and induction of IFN-stimulated genes and transcription factors like IRF-3 and NF-κB [60]. The counteractive mechanisms of HBV that prevent activation of the innate responses are summarized in Table 1.

**Table 1:** Summary of the mechanisms employed by HBV to counteract induction of an efficient interferon response in infected cells

**Experimental model**	**Mechanism**	**HBV protein involved**	**Functional outcome**	**Reference**
**Human hepatocyte cell line**	Inhibition of IRF3	HBV polymerase	Inhibition of IFN-β induction	[52]
**Hepatoma cell lines, liver tumor samples**	MAVS degradation	HBx	Prevention of IFN-β induction	[55]
**Hepatoma cell lines**	Blocking DDX3 DEAD box RNA helicase	HBV polymerase	Inhibition of IFN-β promoter in response to RIG-I/MDA5 and TLR3	[51]
**Hepatoma cell lines, HBx transgenic mouse**	HBx binding to IPS-1	HBx	Inhibition of IFN-β induction	[54]
**Hepatoma cell lines**	Binding and inactivation of mitochondrial membrane protein virus-induced signalling adaptor (VISA)	HBx	Inhibition of virus-triggered IRF3 activation and IFN-β induction	[53]
**HBV transgenic mice and hepatoma cell lines**		HBsAg, HBeAg, or HBV virions	Suppression of TLR 1-9 induced activation of IRF3, NF-κB, ERK1/2 and IFN-β production	[58]
**VSV infection of hepatoma cell lines and monocyte-derived dendritic cells from patients with HBV infection**			Reduced RIG-I expression and IFN-β production	[50]

However, the actual efficacy of these inhibitory mechanisms needs to be established in the context of an in-vivo infection model. The active inhibition of innate immunity may not be very robust, since it can be overcome by exogenous stimulation. In this respect, TLR activation was shown to control viral replication in vivo in HBV transgenic mice [20].

## Conclusions

The role of innate immunity in controlling HBV replication has been neglected for a number of years due to results from studies with HBV infected animals that failed to detect induction of type 1 interferon responses following HBV challenge. The ability of antibody responses to HBV surface antigen to protect adults from HBV challenge also indicated the importance of adaptive immunity in controlling viral replication. However, adaptive immunity alone cannot explain the persistence of HBV during chronic infection and the immune pathogenesis that characterizes chronic hepatitis B. A better understanding of how the immune systems works has lead to a reconsideration of the role of innate immunity in HBV infection. Recent research studies mentioned in this review have shown a different image of HBV pathogenesis, with active mechanisms aimed at inactivating various components of the innate immune system. Thus, the lack of interferon responses during acute infection seems to be the result of inactivation of various signalling pathways that normally induce IFN production in other viral infection. HBx and HBV polymerase are the proteins associated most frequently with inactivation of the TLR and RIG-I pathways and ultimately with impaired IFN production. These mechanisms may constitute important factors of viral persistence during natural infection, since in vitro studies have shown that HBV is sensitive to antiviral properties of IFNs. During the course of infection HBV also contributes to a sustained immunosuppressive state that may favour its replication, since the number of Tregs increase as disease progresses, which also decreases the number and function of HBV specific T lymphocytes.

Other components of the innate immune system that are targeted by the virus are DCs, NK and NKT cells. Their activation during acute infection or immunization is linked to a favourable clinical outcome and subsequent robust adaptive immune responses. By disrupting innate immunity pathways early during infection, the virus also compromises the quality of adaptive immune response, in favour of its survival. The interplay between innate and adaptive immunity for successful control of HBV infection is complex and needs to be addressed in future therapeutic approaches aimed at controlling viral replication.

## Abbreviations

CTLs: Cytotoxic CD8+ lymphocytes; HBV: Hepatitis B virus; HCV: Hepatitis C virus; IFN: Interferon; IL: Interleukin; MDA5: Melanoma differentiation associated gene 5; MHC: Major-histocompatibility-complex; NF-κB: Nuclear factor kappa-light-chain enhancer of activated B cells; NK cells: Natural killer cells; PRRs: Pattern-recognition receptors; PKR: Double stranded RNA-dependent protein kinase; RIG-I: Retinoic acid inducible gene I; TGF-β: Transforming growth factor beta; TL: Toll-like receptor; TNF-α: Tumor necrosis factor alpha; Treg: Regulatory T cell.

## Competing interests

The authors declare that they have no competing interests.

## Authors’ contributions

AB wrote the review, AK edited and critically appraised the manuscript as AB’s supervisor. Both authors read and approved the final manuscript.
